# Direct and indirect associations of childhood adversities with functional impairment and life stress among military personnel

**DOI:** 10.1017/S0033291725101189

**Published:** 2025-07-31

**Authors:** Laura Campbell-Sills, Emily R. Edwards, Sam D. Strizver, Karmel W. Choi, Jason D. Kautz, Santiago Papini, James A. Naifeh, Pablo A. Aliaga, Paul B. Lester, Ronald C. Kessler, Robert J. Ursano, Murray B. Stein, Paul D. Bliese

**Affiliations:** 1 Department of Psychiatry, University of California San Diego, La Jolla, CA, USA; 2 VISN 2 MIRECC, Department of Veterans Affairs, Bronx, NY, USA; 3 Department of Psychiatry, Yale School of Medicine, New Haven, CT, USA; 4 Department of Psychiatry, Icahn School of Medicine at Mount Sinai, New York, NY, USA; 5 Department of Management, Darla Moore School of Business, University of South Carolina, Columbia, SC, USA; 6 Center for Precision Psychiatry, Department of Psychiatry, Massachusetts General Hospital, Boston, MA, USA; 7 Psychiatric and Neurodevelopmental Genetics Unit, Center for Genomic Medicine, Massachusetts General Hospital, Boston, MA, USA; 8 Department of Organizations, Strategy, and International Management, University of Texas at Dallas, Dallas, TX, USA; 9 Department of Psychology, University of Hawaiʻi at Mānoa, Honolulu, HI, USA; 10 Center for the Study of Traumatic Stress, Department of Psychiatry, Uniformed Services University of the Health Sciences, Bethesda, MD, USA; 11 Henry M. Jackson Foundation for the Advancement of Military Medicine, Bethesda, MD, USA; 12 Graduate School of Defense Management, Naval Postgraduate School, Monterey, CA, USA; 13 Department of Health Care Policy, Harvard Medical School, Boston, MA, USA; 14 Herbert Wertheim School of Public Health and Human Longevity Science, University of California San Diego, La Jolla, CA, USA

**Keywords:** adverse childhood experiences, mental disorders, psychosocial functioning, psychological stress, mediation analysis, military personnel

## Abstract

**Background:**

Adverse childhood experiences (ACEs) are associated with physical and mental health difficulties in adulthood. This study examines the associations of ACEs with functional impairment and life stress among military personnel, a population disproportionately affected by ACEs. We also evaluate the extent to which the associations of ACEs with functional outcomes are mediated through internalizing and externalizing disorders.

**Methods:**

The sample included 4,666 STARRS Longitudinal Study (STARRS-LS) participants who provided information about ACEs upon enlistment in the US Army (2011–2012). Mental disorders were assessed in wave 1 (LS1; 2016–2018), and functional impairment and life stress were evaluated in wave 2 (LS2; 2018–2019) of STARRS-LS. Mediation analyses estimated the indirect associations of ACEs with physical health-related impairment, emotional health-related impairment, financial stress, and overall life stress at LS2 through internalizing and externalizing disorders at LS1.

**Results:**

ACEs had significant indirect effects via mental disorders on all functional impairment and life stress outcomes, with internalizing disorders displaying stronger mediating effects than externalizing disorders (explaining 31–92% vs 5–15% of the total effects of ACEs, respectively). Additionally, ACEs exhibited significant direct effects on emotional health-related impairment, financial stress, and overall life stress, implying ACEs are also associated with these longer-term outcomes via alternative pathways.

**Conclusions:**

This study indicates ACEs are linked to functional impairment and life stress among military personnel in part because of associated risks of mental disorders, particularly internalizing disorders. Consideration of ACEs should be incorporated into interventions to promote psychosocial functioning and resilience among military personnel.

## Introduction

Adverse childhood experiences (ACEs), such as exposure to abuse, neglect, or stressful household dysfunction, are common and have harmful effects that persist across the lifespan (Bhutta et al., 2023; Felitti et al., 1998; Madigan et al., 2023; Norman et al., 2012). Research links ACEs to poor health outcomes in adulthood, including high-risk behaviors (e.g., substance misuse; Campbell, Walker, & Egede, 2016; Felitti et al., 1998), physical diseases (Afifi et al., 2016; Felitti et al., 1998), mental disorders and suicidal behaviors (Bruffaerts et al., 2010; Dube et al., 2001; Kessler et al., 2010; Kessler, Davis, & Kendler, 1997), and premature death (Brown et al., 2009). Moreover, affected individuals often report multiple ACEs (Ford, Elhai, Connor, & Frueh, 2010; Kessler et al., 1997), and more extensive exposure to ACEs imparts even greater liability to poor health outcomes (McLaughlin, 2016).

Some young adults may attempt to escape abusive, neglectful, or otherwise difficult home environments by enlisting in the military (Mankowski, Tower, Brandt, & Mattocks, 2015; Sadler, Booth, Mengeling, & Doebbeling, 2004; Woodruff, Kelty, & Segal, 2006). Indeed, epidemiological studies consistently find higher prevalence of ACEs among all-volunteer force personnel relative to civilians (Afifi et al., 2016; Blosnich et al., 2014, 2021; Katon et al., 2015). For example, in one US population-based study, male military personnel from the all-volunteer era had higher prevalence of any ACEs (73% *vs* 58%) and of extensive ACEs exposure (4 or more ACEs; 27% *vs* 13%) relative to male civilians (Blosnich et al., 2014). Differences in ACEs were less pronounced in female military personnel vs civilians; however, females who had served in the military had a higher prevalence of several forms of ACEs, including sexual abuse, physical abuse, and emotional abuse. As in civilians, ACEs are associated with increased risks of mental disorders and suicidal behaviors among military personnel (Afifi et al., 2016; Crede, Tynan, Harms, & Lester, 2023; Moore et al., 2024; Panza et al., 2022; Stein et al., 2018). Given their high prevalence and apparent mental health impacts, a greater understanding of the long-term sequelae of ACEs is one important element of broader efforts to improve health outcomes of military personnel across their service history (Belding et al., 2022; Naifeh et al., 2019; Peterson et al., 2021).

ACEs are conceptualized as having “cascading effects” whereby proximal sequelae introduce additional risks, which further undermine health and well-being (Bhutta et al., 2023; Jones, Nurius, Song, & Fleming, 2018). An implication is that the enduring impact of ACEs may include not only excess risks of specific diseases or disorders but diminished overall functioning and well-being in the longer term. Mental disorders are likely instrumental to these detrimental pathways, given their potential influences on psychosocial functioning and life stress (Hammen, 2005; Ormel et al., 2008). For example, ACEs are implicated in the onset and maintenance of internalizing disorders, such as depressive, anxiety, and trauma-related disorders (Khanijahani & Sualp, 2022). These disorders commonly lead to avoidance and may interfere with motivation to pursue goal-directed behaviors, thereby limiting occupational and social activities and inhibiting the resolution of life stressors. ACEs may also contribute to the development and maintenance of externalizing disorders, such as substance use and intermittent explosive disorders (Khanijahani & Sualp, 2022), which routinely interfere with goal attainment by introducing self-defeating barriers. It is therefore plausible that mental disorders are key mediators of relationships between ACEs and longer-term functional outcomes and that mediation operates differently for internalizing vs externalizing disorders.

A better understanding of the pathways by which ACEs impact functional outcomes in adulthood could inform efforts to promote the health and resilience of military personnel, a population disproportionately affected by ACEs. Investigating the long-term sequelae of ACEs within military samples is vital, given that results from civilian studies of this topic cannot necessarily be extrapolated to service members, whose early adulthood is shaped by factors that could either buffer (e.g., stability/structure of military service) or magnify impacts of ACEs (e.g., exposure to combat or other occupational stressors). The current study uses three waves of survey data from the Army Study to Assess Risk and Resilience in Servicemembers (Army STARRS; Kessler et al., 2013; Ursano et al., 2014) and the STARRS Longitudinal Study (STARRS-LS; Naifeh et al., 2019) to test the hypothesis that ACEs are associated with adult functional impairment and life stress through internalizing and externalizing mental disorders. This prediction was tested by modeling the joint effects of ACEs and mental disorders on subsequent functional impairment and life stress, and by estimating the proportion of the total statistical effect of ACEs mediated through the mental disorder variables.

We also evaluated two corollary hypotheses. First, we expected ACEs to be associated with adult functional impairment and life stress beyond the statistical effects conveyed through psychopathology. Results showing *partial* mediation by mental disorders and incremental (direct) effects of ACEs on the study outcomes would support this prediction. This corollary hypothesis is based on evidence that ACEs are implicated in a range of neurodevelopmental and other biological changes (Beck et al., 2025; Bhutta et al., 2023; Cecil, Zhang, & Nolte, 2020; Gonzalez-Castro et al., 2023; McLaughlin, Weissman, & Bitran, 2019; Nakama, Usui, Doi, & Shimada, 2023), liability to further trauma exposure, and restricted access to social support (Bhutta et al., 2023; Jones et al., 2018). Thus, we deemed it plausible that ACEs might also lead to functional impairment and life stress through biological or behavioral alterations that were not expressed as psychopathology. Second, we expected that internalizing disorders (as opposed to externalizing disorders) would be more influential mediators of the associations of ACEs with later functional impairment and life stress. This corollary prediction was based on evidence of greater role impairment and stress susceptibility associated with internalizing vs externalizing disorders (Kessler et al., 2014; Ormel et al., 2008).

## Methods

### Overview and participants

This study uses baseline data from the Army STARRS New Soldier Study (NSS; Kessler et al., 2013; Ursano et al., 2014) and follow-up data from STARRS-LS (Naifeh et al., 2019). The STARRS research program aims to yield information pertinent to mental health risk reduction and resilience-building for military personnel; as such, STARRS surveys evaluate a wide range of risk and resilience factors (e.g., ACEs, other potential traumas, personality characteristics, service-related factors) as well as mental health, military/career, and functional outcomes. Written informed consent was obtained for participation in the NSS and STARRS-LS surveys and for linkage of survey responses to US Army/Department of Defense (DoD) records. NSS and STARRS-LS procedures were approved by the Institutional Review Boards of the collaborating institutions.

The baseline NSS was conducted from April 2011 to November 2012. Computer-based surveys were administered during soldiers’ first week in the Army, as they were preparing for basic combat training. Details regarding the design and procedures of the NSS are available in prior reports (Heeringa et al., 2013; Kessler et al., 2013). STARRS-LS, which began in 2015 and is projected to continue through 2030, follows a probability sample of Army STARRS (including NSS) participants who agreed to linkage of their survey responses and Army/DoD administrative data. STARRS-LS oversampled Army STARRS baseline survey respondents with a history of mental disorders or suicidality, as well as women, National Guard/Reserve members, and Special Operations soldiers. Earlier reports provide detailed information regarding sampling, weighting, and other STARRS-LS procedures (Naifeh et al., 2022; Stanley et al., 2022).

**Figure 1.**
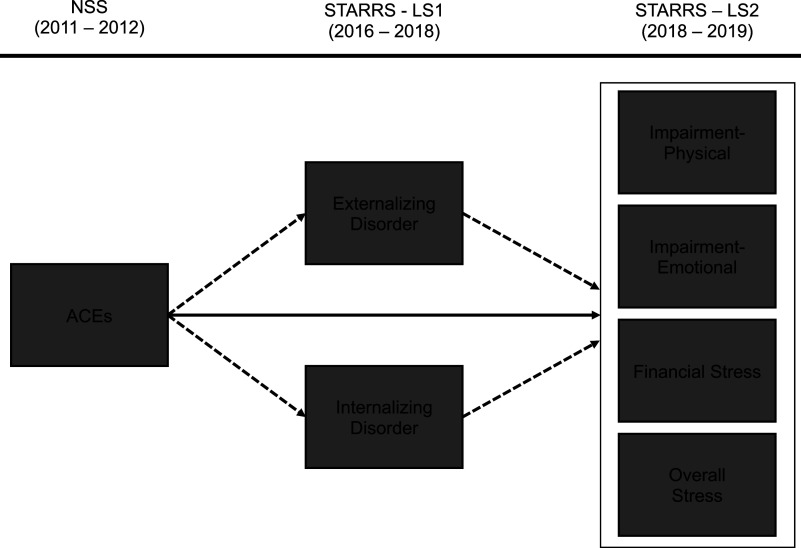
Overview of the study design. NSS = New Soldier Study; STARRS-LS1 = wave 1 of the STARRS Longitudinal Study; STARRS-LS2 = wave 2 of the STARRS Longitudinal Study; ACEs = adverse childhood experiences.

The current study uses data from wave 1 (LS1; 2016–2018) and wave 2 (LS2; 2018–2019) of STARRS-LS to examine outcomes of NSS participants several years after enlistment (Figure 1). Of the 38,507 soldiers included in the NSS (Rosellini et al., 2015), 6,283 were recruited for and completed the LS1 survey (mean time between surveys = 5.78 years, SD = 0.71), and 5,129 also completed the LS2 survey (mean time between LS1 and LS2 = 0.96 years, SD = 0.24). The sample was further restricted to participants who provided information for all measures used in the current study. The final sample (*N* = 4,666) consists of both active-duty (*n* = 1187) and veteran (*n* = 3479) military personnel.

Complex sampling weights were applied in all analyses, which are explained in detail elsewhere (Naifeh et al., 2022). The weights incorporate nonresponse and poststratification adjustments for Army STARRS baseline and STARRS-LS follow-up survey data, as well as adjustments to account for oversampling of baseline respondents with certain characteristics (described earlier) and underrepresentation of difficult-to-recruit participants in STARRS-LS.

### Measures

#### Adverse childhood experiences (ACEs)

The NSS survey assessed the frequency of stressful experiences that occurred before age 18. Experiences queried in the survey were grouped into six categories following prior work (Felitti et al., 1998): psychological abuse, physical abuse, sexual abuse, substance abuse in household, mental illness in household, and criminal behavior in household. To derive an ACEs score, raw item-level responses were first recoded to identify whether respondents were exposed to a specific experience at least once (coded 1) or were never exposed to that experience (coded 0). Next, scores for each of the ACEs categories were derived, with 1 reflecting exposure to at least one experience from that category and 0 reflecting a lack of exposure to experiences in that category. The dichotomous category scores were then summed, yielding an overall ACEs score for each participant ranging from 0 (no ACEs) to 6 (exposure to all six forms of ACEs).

#### Mental disorders

The LS1 and LS2 surveys assessed DSM-5 criteria for mental disorders (American Psychiatric Association, 2013) using items adapted from the Composite International Diagnostic Interview Screening Scales (Kessler et al., 2013; Kessler & Ustun, 2004) and the posttraumatic stress disorder (PTSD) checklist for DSM-5 (Blevins et al., 2015). Previous research suggests good agreement between diagnoses derived from participants’ responses to these items vs clinical interview (Kessler et al., 2013). Prior work also supports a distinction between internalizing and externalizing disorders (Kessler et al., 2011), which was used to categorize the disorders assessed in the LS1 and LS2 surveys. Internalizing disorders included past-30-day generalized anxiety disorder, major depressive disorder, panic disorder, and PTSD, as well as past-12-month mania/hypomania. Externalizing disorders included past-30-day alcohol use disorder, drug use disorder, and intermittent explosive disorder, as well as past-6-month attention-deficit/hyperactivity disorder. Internalizing disorder was defined as screening positive for at least one of the listed internalizing disorders, and externalizing disorder was defined as screening positive for at least one of the listed externalizing disorders.

#### Functional impairment

The LS2 survey included items adapted from the role impairment subscales of a validated measure of health-related quality of life (Hays, Sherbourne, & Mazel, 1995). The items assessed the degree to which physical and emotional issues limited respondents in their activities in the 30 days before the survey. Degree of impairment was rated on a 5-point scale from ‘none of the time’ (coded ‘0’) to ‘all or most of the time’ (coded ‘4’). For the measure of physical health-related impairment (impairment physical), respondents were asked about the frequency at which they accomplished less than they would have liked, were limited in the kind of work or other activities they had access to, and had difficulty performing their work or other activities due to physical health issues. The measure of emotional health-related impairment (impairment-emotional) contained the same questions, with an additional fourth item asking respondents the frequency at which they performed work or other activities less carefully than usual due to emotional issues. Summary scores were derived by averaging the ratings of each item pertaining to impairment-physical (Cronbach’s alpha = .91) and impairment-emotional (Cronbach’s alpha = .94), respectively.

#### Life stress

The LS2 survey asked respondents to indicate how much stress they currently had in each of the following areas of life: finances, career, health, close personal relationships, relationships with family and friends, health of loved ones, other problems experienced by loved ones, problems getting along with others, and ‘life overall’. Respondents rated their stress in each domain on an 11-point scale from ‘no stress’ (coded ‘0’) to ‘very severe stress’ (coded ‘10’). The 0–10 rating of the finances item served as the measure of financial stress, which is a key adjustment factor for military personnel, particularly after separation. The Overall Life Stress score was derived by averaging the ratings of each item (Cronbach’s alpha = .92; note that the reported results are unchanged if the finances item is omitted from the overall life stress score).

#### Potential confounders

Previous STARRS investigations have found sociodemographic variables to be associated with ACEs (Stein et al., 2018) and mental disorders (Kessler et al., 2014; Rosellini et al., 2015); thus, we included these as covariates in all models. Information regarding sex, age, race and ethnicity, and education was taken from the NSS survey, and marital status was obtained from the LS1 survey. Sex was assessed using the question, ‘Are you male or female?’ and coded as a dichotomous variable (1 = Male; 0 = Female). Race and ethnicity were assessed with two survey items (‘What is your race?’ and ‘Are you Spanish/Hispanic/Latino?’); in the current analyses, those classified as non-Hispanic White were contrasted with all others. Education was evaluated using the item, ‘What is the highest level of education you completed?’ and classified as high school degree (high school diploma or GED/equivalent), undergraduate degree, or graduate degree. Marital status was assessed using the question ‘What is your marital status?’ and categorized as married, never married, or other (divorced, separated, or widowed).

### Data analysis

Mediation analyses were conducted using the ‘mediation’ package (Tingley et al., 2014) in R version 4.3.2 (R Core Team, 2024) to estimate the indirect effect of ACEs on impairment-physical, impairment-emotional, financial stress, and overall life stress at LS2 through that of internalizing disorders and externalizing disorders at LS1. Mediation analysis decomposes the total effect of an independent variable on a dependent variable into a direct effect and one or more indirect effects facilitated by additional mediating variable(s). We estimated the average causal mediation effect (ACME) and proportion of the total effect mediated following recommended procedures (Imai, Keele, Tingley, & Yamamoto, 2010). Separate models were tested for each outcome variable, with covariates included. Because internalizing and externalizing disorders may co-occur, both mediators were included when modeling the indirect effects of ACEs to account for the unique mediating influence of each variable on the outcome variables. Mediation models were estimated using logistic regression to model the effects of ACEs on the likelihood of internalizing and externalizing disorders at LS1, as well as ordinary least squares regression to model the effects of internalizing and externalizing disorders at LS1 on each of the outcome variables. Additionally, the mediation analyses applied a quasi-Bayesian Monte Carlo simulation approach with 1,000 iterations to estimate robust effects and obtain 95% confidence intervals for the indirect effects. To test the robustness of the direct associations of ACEs with the LS2 outcomes, a sensitivity analysis was conducted that added controls for internalizing and externalizing disorders at LS2. This included models that incorporated the main effects of LS2 mental disorders as well as interaction effects of LS1 mental disorders × LS2 mental disorders.

## Results

Complete sample characteristics (*N* = 4,666) can be found in Supplementary Table 1; all of the proportions and means are weighted. Briefly, the majority of participants were male (*n* = 3634; 82%), White (*n* = 3066; 63%), and high school-educated (*n* = 3340; 77%), and the mean age at baseline was 21.2 (SD = 3.8). Table 1 shows the distribution of ACEs; approximately half of the participants reported at least 1 form of ACEs and 11% reported 3 or more forms of ACEs assessed in the NSS survey. A total of 24% (*n* = 1015) screened positive for internalizing disorder and 11% (*n* = 447) screened positive for externalizing disorder at LS1. At LS2, participants reported that functional impairment stemming from physical and emotional difficulties occurred ‘a little’ to ‘some of’ the time on average (*M* = 1.74, SD = 0.99 and *M* = 1.76, SD = 1.01, respectively). Financial stress was generally rated as mild to moderate (*M* = 3.41, SD = 2.86), and overall life stress was generally rated as mild (*M* = 2.48, SD = 1.99).

**Table 1.** Adverse childhood experiences reported by new US Army soldiers (*N* = 4666)

Number of ACEs reported	*n* (weighted %)	Type of ACEs reported	*n* (weighted %)
0	2283 (51.4%)	Psychological abuse^a^	1805 (35.9%)
1	1105 (24.1%)	Physical abuse^b^	994 (19.5%)
2	707 (14.2%)	Sexual abuse^c^	397 (7%)
3	344 (6.2%)	*Parental dysfunction:*	
4	168 (2.8%)	Substance abuse^d^	376 (7.2%)
5	55 (1.3%)	Mental illness^e^	586 (11.5%)
6	4 (.07%)	Criminal behavior^f^	364 (8%)

*Note*: ACEs were assessed at baseline in the New Soldier Survey. ACEs = adverse childhood experiences.

a Response of *Rarely, Sometimes, Often*, or *Very Often* to ‘You were emotionally abused at home’ or ‘People in your family said hurtful or insulting things to you’ before age 18.

b Response of *Rarely, Sometimes, Often*, or *Very Often* to ‘You were physically abused at home’ or ‘Someone in your family hit you so hard that it left bruises or marks’ before age 18.

c Response of *Rarely, Sometimes, Often*, or *Very Often* to ‘You were sexually abused at home’ or ‘Someone touched you or made you touch them in a sexual way against your will’ before age 18.

d Response of *1* to *10+ years* to ‘How many years was either of your parents (or the people who raised you) so seriously impaired that it interfered with their parenting, work, or other daily activities because of an alcohol or drug problem?’ before age 18.

e Response of *1* to *10+ years* to ‘How many years was either of your parents (or the people who raised you) so seriously impaired that it interfered with their parenting, work, or other daily activities because of a mental illness?’ or response of *Yes* to ‘A parent committed suicide’ before age 18.

f Response of *Yes* to ‘A parent was in prison or jail for 6 months or longer’ before age 18.

Adjusting for the potential confounders, range of exposure to ACEs was significantly associated with likelihood of screening positive for internalizing disorders (*b* = 0.33, SE = 0.03, *t* = 9.51, *p* < .01) and externalizing disorders (*b* = 0.37, SE = 0.05, *t* = 7.17, *p* < .01) at LS1. Results of models estimating the joint associations of ACEs score and mental disorders with subsequent functional impairment and life stress are summarized in Table 2, with additional details shown in Supplementary Tables 2a–5a. Range of ACEs was significantly associated with emotional health-related impairment, financial stress, and overall life stress at LS2, adjusting for the potential confounders as well as internalizing disorders and externalizing disorders at LS1. Range of ACEs was not significantly related to physical health-related impairment at LS2 after adjusting for the potential confounders and mental disorders at LS1.

**Table 2.** Associations of ACEs with functional impairment and life stress adjusting for sociodemographic factors and mental disorders

	Outcomes at wave 2 of STARRS-LS			
	Impairment-physical	Impairment-emotional	Financial stress	Overall stress
Male (ref: female)	−.07	−.11**	−.39**	−.28**
White (ref: any other race/ethnicity)	−.14**	−.07	−.08	−.10
Age, years	.03**	.00	.02	.02
Marital status at LS1 (ref: married)				
Never married	−.14**	−.04	.06	.03
Divorced, separated, or widowed	.04	.13	.74**	.45**
Education (ref: high school degree)				
College degree	−.10	.00	−.26*	−.02
Graduate degree	−.28*	−.03	−.82**	−.07
Internalizing disorder at LS1	.74**	.99**	2.02**	1.85**
Externalizing disorder at LS1	.08	.24**	.49	.45*
Range of ACEs (0–6)	−.00(.05*)	.04**(.11**)	.23**(.39**)	.15**(.29**)

*Note:* df = 4655 (4657). Standard errors, *t*-values, and *p*-values included in Supplementary Tables 2a–5a. Values in parentheses are from models excluding mental disorders. Unless otherwise noted, the independent variables were collected in the Army STARRS New Soldier Study. ACEs = adverse childhood experiences.

* *p* < .05,

** *p* < .01.

**Figure 2.**
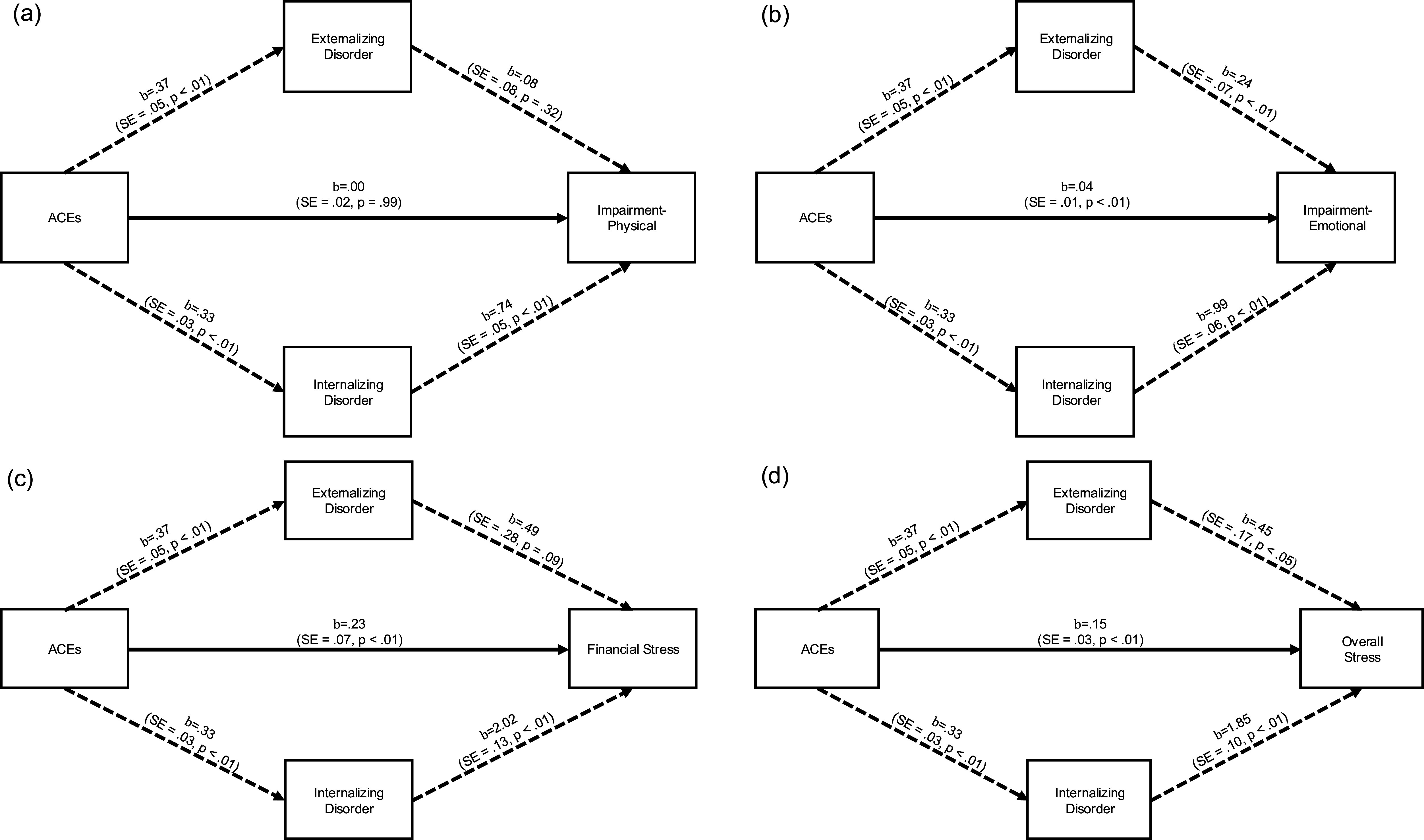
Summary of the observed associations of ACEs and mental disorders with (a) physical health-related impairment, (b) emotional health-related impairment, (c) financial stress, and (d) overall life stress. All estimates are adjusted for the effects of sociodemographic variables. ACEs = adverse childhood experience.

Our study hypothesis (inclusive of the two corollaries) proposed partial mediation whereby ACEs would relate to later functional impairment and life stress via internalizing and externalizing disorders, with an expectation that the mediation effects would be stronger for internalizing disorders. Table 3 summarizes the indirect associations of ACEs with the outcomes and proportions of the total associations mediated by internalizing and externalizing disorders. Consistent with hypotheses, the ACEs score was indirectly associated with all four outcomes through internalizing disorders, with the proportion of the total effect mediated ranging from 31% (financial stress) to 92% (physical health-related impairment; see Table 3). In contrast, ACEs score was indirectly associated with only two of the outcomes through externalizing disorders (see Table 3), with an estimated 15% of the total effect of ACEs on emotional health-related impairment and 8% of the total effect of ACEs on overall life stress mediated through externalizing disorders. The results of all analyses estimating the associations among ACEs, mental disorders, and the study outcomes are summarized in Figure 2.

**Table 3.** Indirect associations of ACEs with functional impairment and life stress outcomes through internalizing and externalizing disorders

	Internalizing disorder at LS1		Externalizing disorder at LS1	
	ACME	Proportion mediated	ACME	Proportion mediated
Impairment-physical	.04**	.92	.00	.05
Impairment-emotional	.05**	.57	.01*	.15
Financial stress	.11**	.31	.01	.05
Overall stress	.10**	.40	.01*	.08

*Note*: The analysis evaluated the extent to which internalizing disorders and externalizing disorders mediated the total effect of ACEs score on functional impairment and life stress outcomes at LS2. ACME = average causal mediation effect. LS1 = wave 1 of STARRS-LS; LS2 = wave 2 of STARRS-LS.

* *p* < .05,

** *p* < .01.

Results of the sensitivity analysis were consistent with the findings of the main analysis. When accounting for internalizing and externalizing disorders at both LS1 and LS2 (and LS1 x LS2 disorder interactions), the direct association of ACEs remained non-significant in all models of physical health-related impairment and significant in all models of emotional health-related impairment, financial stress, and overall life stress (see Supplementary Tables 2b–5b and 6).

## Discussion

This study contributes new information about the long-term sequelae of ACEs. Results indicate that reports of ACEs obtained in early adulthood are associated with later functional impairment and life stress among military personnel, a population disproportionately affected by childhood adversities. Our analysis also shows that the range of exposure to ACEs has both direct associations with functional impairment and life stress, as well as indirect associations with these outcomes through intervening mental disorders. Furthermore, a substantially larger portion of the indirect association of ACEs appears to be mediated through internalizing disorders as opposed to externalizing disorders.

A notable feature of the current study is the prospective design of the mediation analysis. The focal independent variable (range of exposure to ACEs) was measured upon enlistment in the Army, the hypothesized mediators (internalizing and externalizing disorders) were assessed roughly six years later, and functional impairment and life stress were evaluated an additional 1–2 years later. Also noteworthy is the comprehensive survey assessment of mental health, which permits estimation of mental disorder effects absent confounding effects of treatment-seeking. The analyses, which modeled potential mediating effects of both internalizing and externalizing disorders, revealed significant indirect associations of ACEs with all functional and life stress outcomes and significant direct associations of ACEs with three of the four outcomes. These findings imply that ACEs are associated with increased risks of functional impairment and life stress due in part to the psychopathological sequelae that often emerge in the wake of ACEs. Such findings align with other longitudinal studies highlighting the detrimental consequences of ACEs (Afifi et al., 2007; Albott, Forbes, & Anker, 2018; Danese & Widom, 2020, 2023).

When considering internalizing and externalizing disorders together, over 70% of the association of ACEs with emotional health-related impairment was mediated through mental disorders. In the case of physical health-related impairment, mental disorders cumulatively mediated 97% of the statistical effect of ACEs. Mental disorders are therefore key explanatory variables to consider in accounting for the relationship between ACEs and functional impairment in adulthood. The observation of full mediation of the relationship between ACEs and physical health-related impairment by mental disorders was unexpected – and suggests that addressing co-occurring mental health symptoms may be vital to improving functional outcomes among individuals with physical injury/illness and a history of ACEs. Together, mental disorders also explained 36% of the association of ACEs with financial stress and 48% of the association of ACEs with overall life stress. These results implicate psychopathology as a pathway by which ACEs lead to adult life stress – but also suggest that much of the effect of ACEs on subsequent life stress occurs through alternative pathways.

The differentiation of internalizing and externalizing disorders in this study proved to be informative, as the models suggested that internalizing disorders were stronger contributors to the indirect associations of ACEs with the outcomes. Whereas internalizing disorders mediated 31–92% of the total association of ACEs with impairment and life stress, externalizing disorders mediated only 5–15% of the total association of ACEs with these outcomes. While addressing both types of disorders is clinically important, our findings imply that detection and treatment of internalizing disorders – such as PTSD, anxiety disorders, and mood disorders – is particularly important to efforts to improve functional outcomes of military personnel with ACEs. Compared to externalizing behaviors, internalizing symptoms are less apparent to other people; thus, proactive assessment strategies are likely necessary for the detection of these problems.

As explained earlier, we aimed to evaluate fully prospective models that estimated statistical effects of ACEs and mental disorders on *subsequent* functional impairment and life stress outcomes. However, to examine the robustness of our findings, we fit sensitivity models that incorporated the effects of internalizing and externalizing disorders measured *concurrently* with the study outcomes (at LS2). These constituted stringent tests given expectations of strong cross-sectional overlap among mental disorders, impairment, and life stress, which could be magnified by method effects related to concurrent self-report of these domains. Despite this, the direct associations of ACEs with emotional health-related impairment, financial stress, and overall life stress remained significant in all sensitivity models, offering stronger evidence that ACEs are related to functional impairment and life stress independently of mental disorders.

Given that mental disorders explained only a minority of the total effect of ACEs on adult life stress outcomes, other mechanisms underlying these associations must be considered. In this study, financial stress and overall stress were measured with subjective ratings, likely reflecting both the presence/severity of life stressors and respondents’ appraisals of their capacity to manage those circumstances (Epel et al., 2018). Some contexts for ACEs (e.g., maladaptive family functioning, mental illness/substance abuse in family members) may persist over time and lead to elevated levels of adult life stress. Additionally, some biological and behavioral sequelae of ACEs (that are not expressed as psychopathology) may increase the propensity to accumulate life stress or compromise stress management capacity. For example, one investigation found that disruptions to interpersonal functioning associated with ACEs may lead to reduced social support to help manage stressors (Jones et al., 2018). Other research indicates ACEs are associated with altered brain structure and function, with corresponding impacts on physical health, cognitive function, and emotion regulation (Bhutta et al., 2023) – all potential contributors to deficits in stress management and stress proliferation. Future investigations should examine other potential mechanisms by which ACEs impact long-term outcomes, such as adult life stress, with the aim of identifying additional intervention targets beyond mental disorders.

The observed results have direct implications for understanding and reducing functional impairment and life stress experienced by military personnel. Some evidence suggests military service may have a buffering effect against the consequences of ACEs, such that the association between ACEs and functional outcomes is mitigated in military personnel relative to civilians (Katon et al., 2015). Correspondingly, therapeutic considerations of trauma with military personnel tend to emphasize discrete events occurring within the context of service (e.g., combat exposure), and comparatively less attention has been paid to interventions targeting the consequences of ACEs for this population (Carroll, Currier, McCormick, & Drescher, 2017). In contrast to military-based traumas, ACEs occur during sensitive developmental periods and are more likely to be chronic in nature, contributing to a broad range of negative health sequelae as observed in the current study and many others. Because of these differences, interventions to resolve the cascading effects of these forms of trauma exposure also tend to differ. For example, whereas interventions for military-based traumas routinely include immediate, explicit targeting of PTSD symptoms through, for example, cognitive processing of the index trauma (Monson et al., 2006), treatments for trauma experienced within the context of ACEs typically integrate interventions to improve foundational emotion regulation, problem solving, and interpersonal attachment prior to or alongside direct targeting of PTSD symptoms (Cloitre, Cohen, & Koenen, 2011; Harned, 2022). Results of the current study reiterate the likely relevance of this latter approach in the treatment of military personnel with ACEs presenting to care due to concerns of psychopathology, functional impairment, and/or life stress.

Several limitations must be taken into consideration when interpreting the study findings. First, all study variables were measured via self-report, which means the data are vulnerable to response bias. Second, although weights were applied to mitigate the impacts of selection bias and attrition, the study results still may be influenced by these factors. Third, there are various ways to operationalize ACEs, mental disorders, functioning, and stress. Other methods of measuring ACEs (e.g., considering the specific type, frequency, or severity of exposure) or mental disorders (e.g., accounting for the number, severity, or duration of disorders; or using clinical instead of survey-based assessment) could yield different results. Fourth, the surveys did not collect information about the timing of most ACEs; thus, we were unable to consider potential variability in the effects of ACEs based on the age or developmental stage in which they occurred. Finally, although the prospective design allowed for modeling of specific temporal relationships among ACEs, mental disorders, and impairment and life stress outcomes, we cannot assume that longitudinal associations reflect causal relationships. Given the length of the study period, it is likely that many other events and circumstances occurred, and these unmeasured factors could play a role in the reported associations. It is also possible that the direct effects of ACEs on the study outcomes may be partly attributable to subthreshold mental disorder symptoms that were not captured in the diagnostic variables used in the analysis.

## Conclusion

The current study indicates that ACEs are associated with functional impairment and life stress among military personnel, and that these relationships are partly explained by intervening mental disorders. ACEs exhibited indirect associations with all study outcomes through mental disorders, but also had direct associations with three of the four outcomes that were not fully explained by mental disorders. Substantially larger proportions of the indirect associations of ACEs with the study outcomes were explained by internalizing as opposed to externalizing disorders, implying that treatment of internalizing disorders is particularly important for improving functional outcomes of military personnel with ACEs. Consideration of ACEs should also be incorporated into interventions to promote psychosocial functioning and resilience among military personnel. Finally, future research should investigate other mechanisms by which ACEs impact long-term outcomes such as adult life stress, with the aim of identifying additional intervention targets beyond mental disorders.

## Supporting information

Campbell-Sills et al. supplementary material Campbell-Sills et al. supplementary material

## Supplementary material

The supplementary material for this article can be found at http://doi.org/10.1017/S0033291725101189.
