# Supplementary material for: Direct and indirect associations of childhood adversities with functional impairment and life stress among military personnel
Source: Psychol Med. 2025 Jul 31;55:e216. doi: 10.1017/S0033291725101189 (PMC12331149; doi:10.1017/S0033291725101189)
Supplement: Campbell-Sills et al. supplementary material [file S0033291725101189sup001.pdf]

SUPPLEMENTARY MATERIAL

Supplementary Table 1
Sample characteristics (n = 4,666)

	**n (%)**
Age, y, median (range)	20 (18 - 51)
**Sex**	
Male	3634 (82%)
Female	1032 (18%)
**Race and ethnicity**	
White, non-Hispanic	3066 (63%)
Another race or ethnicity	1600 (37%)
**Marital status at LS1**	
Married	2032 (42%)
Never married	2189 (48%)
Divorced, separated, or widowed	445 (10%)
**Education**	
High school degree or GED/equivalent	3340 (77%)
Undergraduate college degree	1215 (22%)
Graduate degree	111 (2%)
**Positive mental disorder screening at LS1**	
Internalizing disorder	1015 (24%)
Externalizing disorder	447 (11%)

Note. Unless otherwise noted, information was collected in the New Soldier Study. LS1 = wave 1 of the STARRS Longitudinal Study.

Supplementary Table 2
Full results of (A) main model and (B) main-effects sensitivity model of physical health-related impairment

A. Main model of the joint associations of ACEs and Mental Disorders with Impairment-Physical

**Variable**	***Est.***	***SE***	***t-value***	***p-value***
Demographics				
Male	-.07	.05	-1.37	.17
White	-.14	.04	-3.41	< .01
Age	.03	.01	3.01	< .01
Marital status				
Never married	-.14	.04	-3.82	< .01
Other	.04	.09	.46	.65
Education				
Undergraduate	-.10	.06	-1.58	.12
Graduate	-.28	.14	-2.08	.04
Internalizing LS1	.74	.05	15.06	< .01
Externalizing LS1	.08	.08	.99	.32
ACEs score (0-6)	-.00	.02	-.01	.99

Note. df = 4655.

B. Sensitivity Model of the joint associations of ACEs and Mental Disorders with Impairment-Physical

**Variable**	***Est.***	***SE***	***t-value***	***p-value***
Demographics				
Male	-.02	.05	-.38	.71
White	-.13	.04	-3.72	< .01
Age	.03	.01	3.35	< .01
Marital status				
Never married	-.15	.03	-4.71	< .01
Other	-.01	.08	-.13	.89
Education				
Undergraduate	-.08	.05	-1.54	.13
Graduate	-.26	.14	-1.84	.07
Internalizing LS1	.40	.06	6.86	< .01
Externalizing LS1	-.04	.09	-.47	.64
Internalizing LS2	.70	.05	13.40	< .01
Externalizing LS2	.22	.07	3.10	< .01
ACEs score (0-6)	-.01	.02	-.59	.56

Note. df = 4653.

Supplementary Table 3
Full results of (A) main model and (B) main-effects sensitivity model of emotional health-related impairment

A. Main model of the joint associations of ACEs and Mental Disorders with Impairment-Emotional

**Variable**	***Est.***	***SE***	***t-value***	***p-value***
Demographics				
Male	-.11	.04	-2.65	< .01
White	-.07	.04	-1.78	.08
Age	.00	.00	.50	.62
Marital status				
Never married	-.04	.05	-.84	.41
Other	.13	.07	1.91	.06
Education				
Undergraduate	.00	.05	.08	.94
Graduate	-.03	.12	-.24	.81
Internalizing LS1	.99	.06	17.04	< .01
Externalizing LS1	.24	.07	3.24	< .01
ACEs score (0-6)	.04	.01	3.04	< .01

Note. df = 4655.

B. Sensitivity Model of the joint associations of ACEs and Mental Disorders with Impairment-Emotional

**Variable**	***Est.***	***SE***	***t-value***	***p-value***
Demographics				
Male	-.03	.04	-.83	.41
White	-.06	.03	-1.93	.06
Age	.00	.00	.81	.42
Marital status				
Never married	-.05	.04	-1.43	.16
Other	.05	.06	.79	.43
Education				
Undergraduate	.02	.04	.54	.59
Graduate	.01	.11	.121	.90
Internalizing LS1	.44	.06	7.61	< .01
Externalizing LS1	.03	.05	.620	.55
Internalizing LS2	1.13	.07	15.52	< .01
Externalizing LS2	.37	.07	5.58	< .01
ACEs score (0-6)	.02	.01	1.99	.04

Note. df = 4653.

Supplementary Table 4
Full results of (A) main model and (B) main-effects sensitivity model of financial stress

A. Main model of the joint associations of ACEs and Mental Disorders with financial stress

**Variable**	***Est.***	***SE***	***t-value***	***p-value***
Demographics				
Male	-.39	.16	-2.46	.02
White	-.08	.10	-.79	.43
Age	.02	.02	1.28	.20
Marital status				
Never married	.06	.13	.50	.62
Other	.74	.17	4.28	< .01
Education				
Undergraduate	-.26	.12	-2.12	.04
Graduate	-.82	.25	-3.28	< .01
Internalizing LS1	2.02	.13	15.97	< .01
Externalizing LS1	.49	.28	1.73	.09
ACEs score (0-6)	.23	.07	3.47	< .01

Note. df = 4655

B. Sensitivity Model of the joint associations of ACEs and Mental Disorders with financial stress

**Variable**	***Est.***	***SE***	***t-value***	***p-value***
Demographics				
Male	-.23	.15	-1.62	.11
White	-.07	.10	-.71	.48
Age	.02	.01	1.59	.11
Marital status				
Never married	.03	.11	.24	.81
Other	.58	.19	3.02	< .01
Education				
Undergraduate	-.23	.11	-2.01	.04
Graduate	-.75	.25	-3.00	< .01
Internalizing LS1	.96	.15	6.48	< .01
Externalizing LS1	.003	.26	.02	.99
Internalizing LS2	2.13	.19	11.08	< .01
Externalizing LS2	.94	.15	6.13	< .01
ACEs score (0-6)	.19	.07	2.75	< .01

Note. df = 4653.

Supplementary Table 5

Full results of (A) main model and (B) main-effects sensitivity model of overall life stress

A. Main model of the joint associations of ACEs and Mental Disorders with overall life stress

**Variable**	***Est.***	***SE***	***t-value***	***p-value***
Demographics				
Male	-.28	.09	-3.32	< .01
White	-.10	.07	-1.39	.17
Age	.02	.01	1.53	.13
Marital status				
Never married	.03	.09	.34	.74
Other	.45	.12	3.74	< .01
Education				
Undergraduate	-.02	.09	.09	.87
Graduate	-.07	.20	.20	.71
Internalizing LS1	1.85	.10	18.39	< .01
Externalizing LS1	.45	.17	2.60	.01
ACEs score (0-6)	.15	.03	4.48	< .01

Note. df = 4655

B. Sensitivity Model of the joint associations of ACEs and Mental Disorders with overall life stress

**Variable**	***Est.***	***SE***	***t-value***	***p-value***
Demographics				
Male	-.13	.07	-1.76	.08
White	-.09	.05	-1.62	.11
Age	.02	.01	2.19	.03
Marital status				
Never married	-.01	.07	-.13	.90
Other	.28	.10	2.76	< .01
Education				
Undergraduate	.02	.07	.31	.76
Graduate	.00	.18	.02	.98
Internalizing LS1	.78	.10	7.67	< .01
Externalizing LS1	-.05	.13	-.38	.71
Internalizing LS2	2.19	.12	17.71	< .01
Externalizing LS2	.96	.11	8.81	< .01
ACEs score (0-6)	.10	.03	3.38	< .01

Note. df = 4653.

Supplementary Table 6
Additional sensitivity models that include interactions between mental disorders at LS1 and LS2

**Variable**	***Impairment-Physical***	***Impairment-Emotional***	***Financial Stress***	***Overall Stress***
Demographics				
Male	-.02	-.02	-.24	-.13
White	-.14**	-.05	-.06	-.08
Age	.03**	.00	.02	.02*
Marital status				
Never married	-.14**	-.05	.02	-.01
Other	-.01	.01	.57**	.27**
Education				
Undergraduate	-.08	-.00	-.23*	.02
Graduate	-.26	-.07	-.75**	.01
Internalizing Disorder LS1	.36**	.37**	1.01**	.72**
Externalizing Disorder LS1	-.15	.07	.18	.10
Internalizing Disorder LS2	.66**	1.09**	2.17**	2.12**
Externalizing Disorder LS2	.14	.40**	1.08**	1.08**
Internalizing LS1 x LS2	.11	.13*	-.12	.13
Externalizing LS1 x LS2	.26	-.06	-.46	.06
ACEs score (0-6)	-.01	.02*	.19**	.10**

Note. df = 4651, $*p < .05$, $**p < .01$.
